# 

**Published:** 1890-11-22

**Authors:** 


### NOTICE TO CORRESPONDENTS.

All MS., Letters, Books for Review, and other matters intended for the Editor should be addressed The Editor, The Lodge, Porchester Square, London, W.

The Editor cannot undertake to return rejected M.S., even when accompanied by stamped directed envelope.

**Notice to Advertisers and Subscribers.**

All Advertisements, Orders for Copies of The Hospital, and Business Communications should be addressed to The Publisher, *not to the Editor*, The Hospital, 140, Strand, W.C.

**Bound Volumes of "The Hospital."**

Vol. VI., containing full page portraits of T.R.H. the Prince and Princess of Wales, and Miss Florence Nightingale, is still obtainable.
Vol. VII. and all the earlier volumes are also for sale.
Orders will be received for Vol. VIII., 4s. post free. Cases for binding may be had of the Publisher, at 1s. 6d. each.

**To Residents, Secretaries, and Matrons.**

The Editor will be glad to have the Regulations and each Report as issued by Asylums, Hospitals, Convalescent and Nursing Institutions, as well as those of other Charities, and an early copy in duplicate of all Appeals and Festival Notices, etc., addressed to *The Editor, The Lodge, Porchester Square, London, W.* Any superintendent, secretary, matron, or other chief official who regularly sends such information may be registered, on application to the Publisher, to receive free of cost a cover for binding The Hospital in half-yearly volumes.

### BURDETT'S HOSPITAL ANNUAL FOR 1890

May be obtained of the Publisher, The Hospital Offices, 140, Strand, W.C., price 2s. 6d. limp boards, or 3s. 6d. cloth. All orders must be accompanied by a remittance.

### Scraps and Gleanings.

Mr. Bernard Wake has contributed £6,000 towards a new wing for the Sheffield Public Hospital.

St. Gabriel's Hospital for Infants is to be amalgamated with the Victoria Hospital for Children.

A strong committee has been formed at Herne Bay to carry out the proposed Cottage Hospital scheme.

The Town Clerk of Oxford is going to replace the £2,000 embezzled from charity moneys by a clerk who has absconded.

Mr. John Cory and Dr. Edwards have in two days collected no less a sum than £1,800 for the debt on Cardiff Infirmary.

Barnsley raised £824 on Hospital Sunday, but is still keeping the books open through a laudable desire to reach £1,000.

The Derbyshire Children's Hospital treated 316 in-patients and 2,018 out-patients last year; the ordinary expenditure was £670.

The British Home for Incurables elected 10 inmates last week. Mr. F. A. Bevan presided, and remarked that funds were very low.

In Madrid last week there were 483 cases of small-pox and 202 deaths. During October there were four deaths from cholera at Madrid.

There was a Fire at Homerton Fever Hospital on Monday, but luckily no lives were lost. It was supposed to originate in the drying closet.

Mr. E. Capper Robson, for twelve years Chairman of Sunderland Provident Dispensary, has been presented with an illuminated address.

The Bishop of Derby preached an eloquent sermon in aid of the Infirmary on November 9th. He was eagerly listened to by a large congregation.

Cardinal Manning has endowed a "Thames Bed" in the London Hospital with the money sent him by the Dockers when he celebrated his jubilee.

High Wycombe Cottage Hospital treated 119 in-patients last year, a very creditable work. The Rev. R. Chilton presided lately at the annual meeting.

Mr. Lawson Tait has been giving evidence at Birmingham in favour of special hospitals. The committee on Hospital Reform met again on November 12th.

North Ormesby Cottage Hospital is to be enlarged at a cost of over £1,000. The new departments will be for the out-patients, and the medical and nursing staffs.

Mr. Fletcher has again tried to enlarge the Quarterly Court of the Radcliffe Infirmary, and has again failed. Mr. Fletcher's charges are too vague to excite real commotion.

Mr. Richard Cadbury's gift of Moseley Hall as a convalescent home for women and children was met with practical difficulties, and it is now agreed that it be used for children only.

Messrs. Hamilton and Co., of Wandsworth, have been awarded a silver medal for their "Pineotas" disinfectant, which has been used successfully throughout the Edinburgh Exhibition.

The Royal Free Hospital issues a special appeal for £5,000, wanted immediately. Some 30 beds are unoccupied for want of funds. The office of collector is abolished, and all subscriptions should be sent to the Secretary.

Collections in aid of certain of the Dublin hospitals took place on Sunday, November 9th. Last year the total amount collected was £4,155 5s. 4d., being an increase of £58 15s. 1d. as compared with the previous return.

The Animals Institute offers prizes for the best regulation collars and badges for dogs, the special features being easy recognition and identification, whilst causing no annoyance to the dog. They must be simple, light, secure, but not costly.

Nottingham General Hospital has held its 108th anniversary. There was service at St. Mary's Church, followed by a general meeting. The in-patients had been 921, and the out-patients 3,293 during the last year. Dr. W. B. Ransom was elected physician.

According to the evidence before the Mansion House Council, the sanitary department is not strong enough to deal with all the evils of Shoreditch. In spite of repeated complaints there are still 282 cases of no water-closet supply, and 57 cases of no dust bins.

Dr. Richardson told some wondrous tales lately of the benefits to public health inaugurated by the late Sir Edwin Chadwick. One tale of Dr. Richardson's was that the sewage of London used to be sent to Jamaica in barrels and these identical barrels came back full of sugar.

A convalescent home for women and children has been dedicated at Otham, near Maidstone. It consists of two large wards with four beds in each, two single wards, a sitting-room, and a matron's room, each 23 ft. 6 in. by 16 ft. 6 in. The building was designed by Mr. Richard Creed, of Finsbury-circus.

The household goods of twenty-six persons distrained upon for refusing to have their children vaccinated were sold by public auction in Stalybridge Market Place. There was considerable indignation during the sale. A meeting was afterwards held to protest against compulsory vaccination, and copies of the Vaccination Acts were publicly burned on the market ground.

At Buda Pesth a painful impression has been created by the death of one of the judges of the Royal Court. The deceased was suffering from acute pains in the stomach, for which the doctor ordered injections of ether. The remedy had scarcely been applied when symptoms of poisoning appeared, and the judge died. Almost immediately the apothecary's assistant appeared saying that he had mistaken the bottle and sent the wrong drug.

The French Academy of Medicine continues to occupy itself with the French depopulation question, and, according to the paper read to them by Dr. Brouardel, one of the most distinguished of specialists, 30,000 deaths occur each year in France which might be easily prevented. It is recommended that a large number of works of sanitary reform be undertaken, which it is anticipated would have the effect of diminishing the extent of the diseases to which this number of deaths is attributed.

### The Student of Medicine.

[All communications for this Supplement should be addressed to The Editor of The Hospital, at 140, Strand, with the words, "Students' Column," written in the left hand top corner of the envelope.]

### EDITORIAL NOTES.

Football.—Although the football season is yet young, and it is perhaps somewhat early to speak decidedly upon the merits of the various hospital teams, we have received sufficient indication to enable us to form a pretty good judgment as to the respective strength of the most prominent of them. Under the Rugby code there is little doubt that St. Thomas's will with ease maintain their premier position. Although they have lost several of their most reliable players, the places seem to have been well filled by new material. Their close matches with Blackheath and Cambridge University, and their crushing defeat of the powerful Richmond club by two goals and three tries to nil, stamp them as one of the strongest southern combinations. Of the other hospitals, Guy's and Middlesex have been most prominent so far, the former drawing with Kensington and making a close fight with Swansea, whilst the latter have defeated several fair teams and made a slightly better match with Croydon than Guy's. Under Association rules, Bart.'s take an easy lead, and are a really first-class team. Their hollow victory over the rangers in the London cup ties by eight goals to love, their defeat by the invincible Royal Arsenal by the narrow majority of one goal, and their score of seven goals to two in an unfinished match against a strong team of casuals are good proofs of their quality. Thomas's, London, and Middlesex all have fair teams, but so far have not done anything of note.

The Ninth of November.—A few years ago this was considered a great day among students, and always formed an excuse for attempting to get up a row; at least this is what we were always led to believe. For instance, it was the general thing on the following day to read in the papers of medical students who were up for various acts of frivolity before the defenders of the peace. We, however, also remember a short time ago, someone took the trouble to go into the various cases that were reported at the police courts, and, speaking from memory, we do not believe that a single one of the accused turned out to be a genuine medical student; but they were young men of various occupations, chiefly in some of the larger houses of business. Having got into trouble, it was a simple matter when asked for a name and occupation to say Smith, of ——— Hospital, a small fine was imposed, and the matter ended; if, on the other hand, the individual said Jones, of Messrs. ———, of Regent Street, the matter did not end, but the culprit in question was speedily discharged from the employment of Messrs. ———, of Regent Street. This state of affairs, which for a long time was not recognised by the authorities, brought many unwarranted charges on the heads of the medical students; so much so that we have even read in the papers of young men being had up and described as of the 'medical student type.' We, however, no longer read of this designation in the columns of the same papers as formerly.

Town and Gown.—The University students also have their day on which to let off their youthful spirits, namely, the 5th of November, on which what used to be known as the "town and gown" rows always occurred. But this has almost ceased at Oxford and Cambridge, and is left to a few freshmen who, from year to year, attempt to keep up the traditions of their ancestors; but it usually terminates in a very feeble exhibition as far as numbers are concerned.

Women and the College of Surgeons.—We publish this week a letter from a correspondent writing from Bartholomew's, and much regret we are unable to answer the question from any scientific standpoint. It is a fact which we cannot explain any more than we can explain a still more curious inconsistency, namely, that the British Medical Association consists of some 16,000 members, and one of these is a lady. Her position is unique; we must accept it, but cannot explain it. Possibly, as in the former inconsistency, tradition has something to do with it; for women were inferior beings to our forefathers. Surely this should be sufficient for us; we would rather not argue the matter; inferior they were, and therefore must be suppressed; we live on tradition, and are hard to teach.

Medicine at Oxford.—Sir Henry Acland, in the *Times*, replies to Mr. Case's letter on the subject of women studying medicine in Oxford, which we noticed last week. He writes: "Why admit them at all? Let them go elsewhere. Twenty-five years ago this language might have sufficed. Women have recently shown in mathematics, in physical science, and in classics that they have attainments equal to those of the best University 'honours' men. They prize the University testamur, though they do not obtain a degree. If women study medicine, as some do, and wish to have the testamur of an old University, why should they not have it, as well as the testamur in classics and mathematics?"

Westminster Hospital.—The annual students' dinner of this hospital was held at the Criterion, on Saturday, November 15th. Dr. Grigg presided. There was a large gathering of past and present students, Dr. P. S. Abraham, and Mr. J. Black being also there. Mr. Vitch Smith distinguished himself both in his oratory and in his musical performances. On the whole the gathering was a great success.

### APPOINTMENTS.

At the North-West London Hospital, H. Dunn, M.B., B.A., C.M., has been appointed medical officer, and C. H. Fowler, M.R.C.S., L.R.C.P., assistant medical officer.—At the Middlesex Hospital, H. G. Morris, L.R.C.P., M.R.C.S., has been appointed house physician.—At the National Dental Hospital of London, Stanley Read, L.D.S., has been appointed house surgeon.—At the Evelina Hospital for Sick Children, Walter B. Dejersey, B.A., M.B., B.C., has been appointed house surgeon.—At the Great Northern Central Hospital, Percy Templeton, M.R.C.S., L.R.C.P., has been appointed house physician.—At the Eastern Dispensary, Liverpool, Albert E. Booth, M.B., C.M., has been appointed house surgeon.—At the Norfolk and Norwich Hospital, W. F. Chomerly, M.R.C.S., L.R.C.P., house surgeon.—At the Lincoln County Hospital, T. Hugh Dickson, M.A., M.B., B.C., M.R.C.S., L.R.C.P., house surgeon.—At the Bradford Infirmary and Dispensary, J. Larcy Frith, M.D., M.R.C.S., L.R.C.P., house physician.—At the Southern Dispensary, Liverpool, E. P. Manby, M.B., B.C., M.R.C.S., house surgeon.—At the Cumberland Infirmary, Carlisle, Louis E. Stevenson, M.B., house surgeon.—At the East London Hospital, Shadwell, H. N. Edwardes, L.R.C.P., M.R.C.S., house physician.—At the Flintshire Dispensary, W. Harrop Parry, M.B., C.M., house surgeon.

### STUDENTS' MEDICAL SOCIETIES.

### The Middlesex Hospital Medical Society.

Thursday evening, November 13th, was devoted to the reading of clinical cases, Mr. A. Pearce Gould in the chair. The following papers were read: "Two Cases of Acute Gastro-Enteritis," by Mr. R. J. Gladstone; "Case of Suppurating Dermoid of Ovary," by Mr. H. S. Coverntow; and a "Case of Supposed Rodent Cancer," by Mr. W. A. Ward. Histological and pathological specimens having reference to the digestive system were shown, also two specimens of rodent cancer.

At the next meeting (Thursday, November 27th), Mr. J. W. Williams will read a paper on "Health in its Relation to Morals."

### Royal Veterinary College Medical Association.

Patron.—Professor G. T. Brown, C.B.

President.—Professor John Penberthy.

Vice-Presidents.—J. R. Cox, F. T. Oatway, W. Pritchard, S. J. Raymend, J. W. Reynolds, H. Sessions, A. Smith, T. Spencer, R. C. Tayler, F. W. Wragg, R. Withers, P. C. Woolston.

Treasurer.—Professor E. S. Shave.

Secretary.—Professor J. McQueen.

Assistant-Secretary.—J. H. Taylor.

The 135th ordinary meeting of this Association was held on November 12th, at half-past six p.m., the president being in the chair, and there were 64 members and 9 visitors present. Mr. J. H. Manton read a most interesting and instructive paper on "Parturient Apoplexy," in which he treated the subject under the following headings: Etiology, Semeiology, Treatment, Prognosis, Post-mortem Appearances, Complications, Sequelæ, and Preventive Measures. The subject was described in a very practical way, and a lively discussion followed, in which the President, Messrs. Spencer, Abrams, Bobett, Wall, Sessions, Sadler, Sullivan, Low, East, Reynolds, and Evans took part.

### THE STUDENTS' BOOKSHELF.

We have received a manual of dental education by Mr. Arthur Turner, published by Messrs. E. and S. Livingstone, of Edinburgh. The whole course best adapted to the use of the intending dental student is fully and clearly set forth in this little book. The introductory note commences with the question, "What shall I be?" and ably points out all the advantages to be derived from the profession, and then dwells upon many of the especial qualities necessary for a high position in the dental profession. A fondness for mechanics is stated to be above all the most important, "for if the student does not possess this he will be severely handicapped, and had better turn his attention to some other occupation." After giving a summary of

### The Student's Bookshelf.—(*Continued.*)

the cost of education, together with many valuable hints as to the preliminary career, Mr. Turner proceeds to draw up a table for the professional curriculum, and he says the best way to arrange a five years' course is as follows: First three years mechanical apprenticeship, fourth and fifth years dental and general hospital courses concurrently. If the student is going to take a medical diploma, these two years will count as two of the four required. A description is then given of the various medical schools for dental students, examinations and medical societies, and a special paragraph is devoted to the consideration of the lady dentist. A few questions of dental etiquette and legal responsibilities are mentioned, and on the question of the giving of anæsthetics, the author states that "there is nothing either to forbid or to allow him to do so." After giving a list of the most useful text-books and a few remarks upon registration, he concludes his excellent little guide with a few specimens of examination papers and oral questions. As a concise epitome of the subject it will be found of great use to those to whom it is addressed.

### LETTER TO THE EDITOR.

[The Editor does not hold himself responsible for opinions expressed by his correspondents.]

WOMEN AND THE COLLEGE OF SURGEONS.

SIR,—I should like to point out a peculiar inconsistency on the part of the College of Surgeons, or, perhaps, I should rather say one of their many peculiar inconsistencies. I refer to the fact that although the College does not allow women to present themselves for examination, it nevertheless reserves its museum for their use one day in the week. If the College approves of medical education for women, it would surely open its examination to them; but if not, why does it offer them such valuable facilities as the museum affords?—Faithfully yours,

St. Bartholomew's Hospital. QUEROR.

### PASS LISTS.

#### Royal College of Surgeons, London.

The following gentlemen passed the first professional examination for the diploma of Fellow at a meeting of the Board of Examiners on the 10th inst.: Albert John Chalmers and John Evans, of University College, Liverpool; Alfred William Hughes, of Edinburgh University; Thomas Whittaker Iddon, of Owens College, Manchester; Malcolm Langton Hepburn, of St. Bartholomew's Hospital; Charles Plumley Childe, of King's College; Ralph Bodkin Mahon, of Dublin; and James Johnstone, of Otago and Aberdeen Universities. Eight candidates were referred.

Passed on the 11th inst.: Frank P. S. Cresswell, of Guy's Hospital; Blackwell Charles, of St. Bartholomew's Hospital; George D. E. Jones, of Middlesex Hospital; Edw. Deansley, of University College; Archibald McKenzie, of Edinburgh University. Eleven candidates were referred.

Passed on the 12th inst.: Henry Rochfort-Brown, student of Oxford University and St. Bartholomew's Hospital; and William Robert Smith, of King's College Hospital. Fourteen candidates were referred back to their professional studies.

#### Society of Apothecaries of London.

The following candidates passed the first examination in chemistry, materia medica, botany, and pharmacy: P. H. Best, B.A. Cantab, Cambridge University and University College; A. H. A. Boyle, Royal Free; A. Harding, Royal Free; E. J. Dodson, Royal Free; S. E. H. Martin, Royal Free.

The following passed in materia medica, botany, and pharmacy: M. E. Bowlby, Royal Free.

The following passed in pharmacy: H. Greenwood, London; J. K. H. Smyth, St. Mary's.

The following passed the second examination in anatomy, physiology, and histology: K. Anderson, St. Bartholomew's; R. E. T. Ingram, Guy's; H. C. Cadman, Owens College, Victoria University; A. N. V. Johnson, Royal Free; R. H. Calvert, Yorkshire College, Leeds, and Durham University; T. L. Johnston, Edinburgh University and University College; H. W. Clarke, St. Mary's; F. B. Lewis, London; H. F. Ealand, St. Mary's; B. J. Macaulay, Middlesex; J. Edwards, London; D. K. M'Dowell, St. Thomas's; A. C. Fenn, St. Bartholomew's; R. E. Nichols, St. Mary's; S. Firth, Yorkshire College, Leeds, and Anderson's College, Glasgow; E. B. Pellatt, Royal Free; C. F. Graves, St. Mary's; J. T. T. Ramsay, Royal College of Surgeons, Edinburgh; S. F. Hogg, Charing Cross.

The following passed in anatomy: W. A. King, Charing Cross.

The following passed in physiology and histology: A. H. Grace, Bristol Medical School; W. Woods, Yorkshire College, Leeds; E. Grange, Edinburgh University.

The following passed the examination in surgery: P. C. De Wet, St. Thomas's; W. S. Whitcombe, Glasgow University; J. R. Fuller, St. Mary's; E. E. Wilbe, St. Bartholomew's; H. W. John, Guy's; W. Wright, St. Bartholomew's; P. A. Shore, Queen's College, Birmingham; W. S. Wright, Bristol Medical School and St. Mary's; A. M. Van Ingen, L.M.S. Madras University.

The following, having previously passed in medicine at this hall, received the diploma of the society qualifying for registration: J. R. Fuller and H. W. John.

The following passed in medicine, forensic medicine, and midwifery:

**Pass Lists**—(*continued*).

T. H. Adams, London; H. L. Morgan, Westminster: J. M. Bennett, Liverpool and Sheffield School of Medicine; C. E. Reinhardt, London; A. B. Blomfield, London; N. A. A. Trenow, St. George's; M. B. Dumaresq, London; A. M. Van Ingen, L.M.S. Madras University; R. A. Irvine, Royal University of Ireland and Glasgow University; F. W. Welstead, Guy's; E. W. Livesey, St. Thomas's; W. S. Whitcombe, Glasgow University; R. H. Mackay, Aberdeen University.

The following passed in medicine and midwifery: G. Wilkinson, Cambridge University and Middlesex.

The following passed in midwifery: E. A. R. Covey, St. Bartholomew's.

The following, having previously passed in surgery at this Hall, received the diploma of the society qualifying for registration: A. B. Blomfield, C. E. Reinhardt, M. B. Dumaresq, N. A. A. Trenow, E. W. Livesey, A. M. Van Ingen, H. L. Morgan, and W. S. Whitcombe.

### ATHLETICS.

### Football.

Mr. G. Munro Smith, in proposing "The Bristol Medical Cricket and Football Clubs, and Kindred Institutions," at the annual dinner of the British Medical School, made an eloquent defence of football. He ridiculed the idea that it was a dangerous game if properly played, and said that all the dangers were due to the caddish conduct of certain players, who forgot that they were men, much less gentlemen, and so used their brute force to maim and play havoc with their fellows. Football, when properly played, was not more dangerous than cricket or athletics, and this view was borne out by the experience of Dr. Clement Dukes, who had held the post of surgeon for more than twenty years to Rugby School, the birthplace and centre of football.

Association Rules.—On November 15th.

*Oxford University v. Old Foresters.*—The first half of the game was well contested, each side scoring a goal, by Roles and Robertson. In the second half, Oxford had much the best of the play, and added six goals to the score, four by Hansard, one each by Wilson and Montgomery. Owing to the lateness in beginning, the game was continued almost into darkness. Final score, Oxford, seven goals; Old Foresters, one. Sides:—Oxford University: E. M. Samson (Trinity) (goal), A. W. Malden (Queen's) and G. M. Hall (St. John's) (backs), R. M. Montgomery (St. Catherine's), E. Church (Keble), and H. A. Tapsfield (Magdalen) (half-back), G. L. Wilson (Hertford) (captain), and W. E. Gilliatt (Magdalen) (left wing), H. A. Rhodes (Church) (centre), G. Reeve (Trinity), and H. Hansard (Oriel) (right wing). Old Foresters: L. Dashwood (goal), F. R. Pelly and S. W. Borrow (backs), E. Horner, E. S. Dashwood and G. T. Hollington (half-backs), R. D. Hodgson, W. Pascoe, A. Robertson, R. C. Guy, and E. B. Alexander.

*Cambridge University v. Corinthians.*—This match was played at Kennington Oval before a large number of people. Cottrill kicked off; the Corinthians at once attacked, and Sandilands scored a goal. Then Cottrill scored a goal, which was disallowed, and Veitch made the score even. Sandilands twice scored for the Corinthians and Gosling for Cambridge. Finally the University won by four goals to three.

*Clapham Rovers* beat *St. Thomas's Hospital*, at Wandsworth, by seven goals to two. *Upton Ivanhoe* played a drawn game with *London Hospital*, each side scoring two goals. *St. Bartholomew's Hospital* beat *Old St Mark's*, by five goals to one. *Clapham Rovers* beat *St. Thomas's Hospital*, by seven goals to two, at Wandsworth Common. *Edinburgh University v. Haddington Wanderers.*—One 'Varsity man being hurt, had to retire, and Haddington won by six goals to one.

Rugby Rules—November 10th.

*Oxford University v. Bradford.*—In this match, which was played on the Merton Ground at Oxford, the University sustained their first defeat this season. The first ten minutes play was all in favour of the Yorkshire team, who obtained two tries through the instrumentality of White and Noble, but the place kicks were not successful. Oxford then played better; but neither side could score, Bradford leading at the interval by two tries to nil. The second half was very keenly contested; but nothing further was added to the score, and the Northerns won a match by two tries to nil.

November 12th.

*London and the South v. Oxford and Cambridge.*—The decisive reverse sustained by London and the South of England at Richmond was to some a great surprise. Although the Southern Rugby Union executive had chosen a very strong fifteen, it was with the greatest ease that the Universities were victorious. Storey kicked off for the Universities, who followed well up, and there were soon scrimmages in the home quarters. The visitors played so well together that from the first they were able to keep the game in their rivals' quarters. Wotherspoon managed to get through after a throw in from touch, and so gained the first point for the Universities. After a very uneven match Oxford and Cambridge were victorious by three goals and two tries to one goal. Sides: London and South—A. E. Stoddart, Blackheath (captain), C. A. Hooper, Middlesex Wanderers, and A. L. Brooke, Old Leysians, three-quarter backs; A. S. Johnstone, Blackheath, back; W. R. M. Leake, Harlequins, and F. C. Duckworth, Somerset, half-backs; G. L. Jeffery, Blackheath, A. A. Surtees, Harlequins, W. A. Morris, Old Edwardians, R. D. Budworth, Blackheath, F. Evershed, Burton-on-Trent, J. E. Aldridge, F. Somes, Somerset, J. H. Rogers, Moseley, and D. W. Helps, Croydon, forwards. Oxford and Cambridge—G. M'Gregor, Jesus, Cambridge, back; P. H. Morrison, Trinity Hall, Cambridge, C. J. N. Fleming, Queen's, Oxford, and P. R. Clauss, Keble, Oxford, three-quarter backs; R. F. de Winton, Exeter, Oxford, and W. Wotherspoon,

**The Hospital Advertiser.**

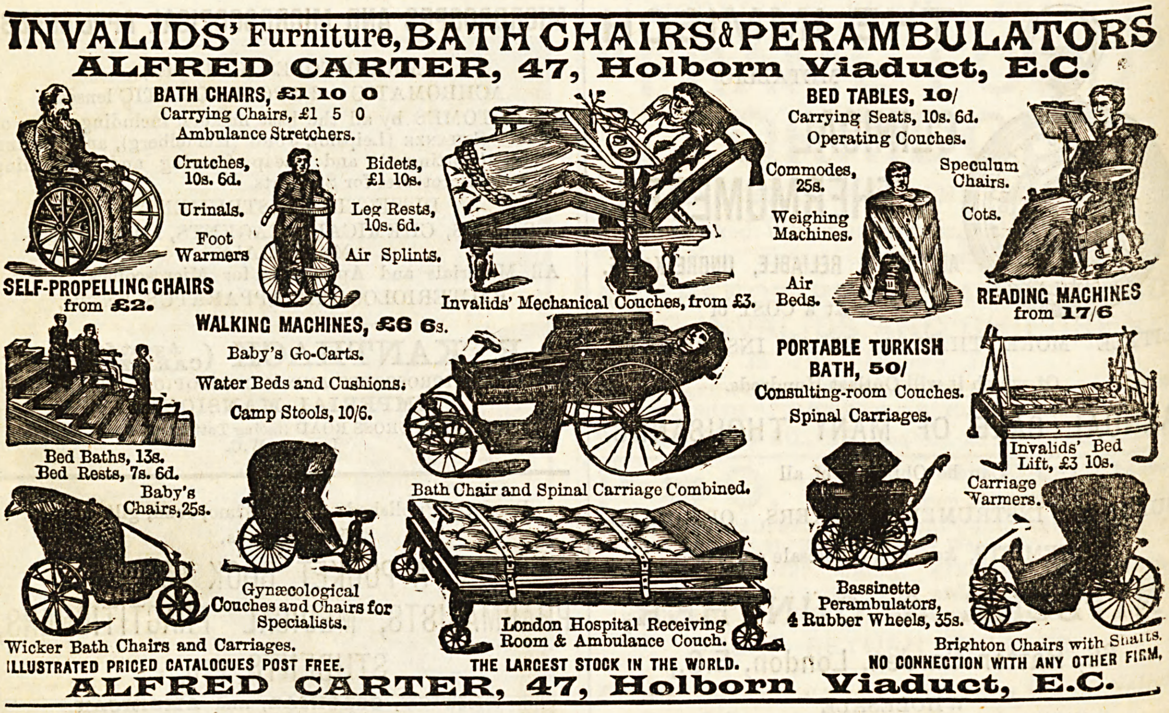

Silks, Dresses, Mantles, Costumes &c.
**Peter Robinson.**
Oxford St. Regent St.

**Keen's Mustard**
The Oldest Manufactory in the City of London.

### Athletics—*continued.*

Clare, Cambridge, half-backs; E. H. G. North, Keble, Oxford, J. H. G. Wilson, Queen's, Oxford, A. R. Kay, Oriel, Oxford, E. Bonham Carter, New, Oxford, L. J. Percival, Trinity, Oxford, F. C. Bree-Frink, St. Peter's, Cambridge, J. C. M'Donnell, Pembroke, Cambridge, T. W. P. Storey, Trinity Hall, Cambridge, and R. Thompson, Pembroke, Cambridge, forwards.

November 13th.

*Guy's Hospital* was beaten by a team of the *University* at Cambridge by five goals and three tries to nil.

November 15th.

*Middlesex Hospital v. Croydon.*—This match was played at Croydon before a large number of people. In the first half of the game Middlesex had the best of it, pressing their opponents continually, but were unable to score, the Hospital forwards playing well, especially in the loose. At half time nothing had been scored by either side; shortly afterwards Marshall, who had been playing up well, met with an accident while tackling an opponent, and was rendered almost useless. Croydon then scored three tries in rapid succession, one of which was converted into a goal. The Hospital then played up splendidly, and after some clever dribbling, Williams secured the ball and ran in. From this point the Hospital pressed their opponents hard, and at the call of time they looked very much like scoring again, being on the Croydon goal line. A splendid game thus ended in favour of Croydon by one goal and two tries to one try. For Croydon, Kelsey, Helps, Hubbard, and Flint played well; and for the Hospital Nicholson, Lee, Beachcroft, and Boazman did excellent service.

*Cambridge University v. Richmond.*—This match was played on the Corpus ground at Cambridge, in most delightful weather. J. E. Biggs obtained a penalty goal for the visitors from a free kick off side. Then Cambridge went ahead, and Wotherspoon placed a goal from a try by Rotherham. At half-time, Cambridge led by three points to two. Finally the University won by twelve points to five.

*King's College v. Dental Hospital.*—This match was played at Wormwood Scrubbs. King's won by two goals and one try to a try. Tries by Penny, Mawson, and Russel for King's, and by Mansell for the Dentals. Goals kicked by Penny.

*Oxford University v. Blackheath.*—Played at Oxford, before a large assembly of people. Fleming kicked off. The University had much the best of the game all through, keeping the ball constantly in their opponents' quarters. Wilson kicked a goal just before half-time from a penalty kick. The match ended by Oxford winning by two goals to nil.

*St. Bartholomew's Hospital v. Cooper's Hill.*—This match was played on Saturday at Cooper's Hill in splendid weather. Boyle secured a try within the first minute, and another two minutes later. Neither were converted into goals. Although the hospital played their best, they were unable to break through. Just before time was called Boyle got in again, the home team thus winning by one goal and two tries to nil.

*Hampstead (Second)* beat *King's College Hospital*, at Wormwood Scrubs (1 t.—1 m.). *St. Bartholomew's Hospital* and *Croydon*, at Balham, drawn (0—0). *Rosslyn Park* beat *Guy's Hospital*, at Acton (2 t.—0). *Old Paulines* beat *University College Hospital*, at Chiswick (1 g., 1t.—0). *Old Leysians* beat *London Hospital (Second)*, at Blackheath (2 g., 2 t.—1 g.). *London Hospital* beat *Upper Clapton*, at Clapton (2 t.—0).

*Westminster Hospital v. 1st Surrey Rifles.*—This match was played at Raynes Park, and resulted in a victory for the medicals by 1 goal, 3 tries to nil. Tries by F. G. Witham, who obtained two, and G. Norman and A. Bell one each. The goal was a magnificent kick by Denne from the touch line.

*Edinburgh University* met the *West of Scotland* on November 15th at Corstorphine. The West scored two goals and the University none. The home team played up well, but were overmatched by the superior weight of their opponents. Of the University, Pender, Donovan, Marsh, and Mitchell deserve special mention. The University team was: T. Dunlop (back), L. D. Young, J. Marsh, J. J. Mitchell (half-backs), J. Pender, G. S. "Thom" (quarter-backs), K. Cameron, W. Davis, R. D. Stokes, Scott, W. Menzies, T. M. Donovan, H. G. Lewar, R. Johnstone, and H. Theodore (forward). *University (Second)* versus *West of Scotland (Second).*—The West won by two goals one try to one try. *Royal College of Surgeons* versus *Grange.*—Grange won by one goal to one try.

### United Hospitals H. and H.

The run with the Hampton Court Club took place on Saturday, the 15th, from the headquarters of the United Hospitals H. and H. at Raynes Park. The trail was laid by G. F. Holt and W. B. Mercer (St. Bartholomew's Hospital), over the Challenge Cup Course, of about nine and a-half miles. The line of country was briefly as follows: Starting from headquarters over a mile and a-half of plough fields into the Merton Road, turning to right into Morden, then bearing to right across heavy plough land into Sutton, and round by Worcester Park, then on into Old Malden, crossing the water jump twice, then making for New Malden, finishing up with a good mile of road by the side of the railway and into Raynes Park. The order of coming in was as follows: (1) F. F. Easton (St. Mary's), (2) G. H. Sydenham (H.C. H. and H.), (3) A. N. Wilde (Barts), (4) A. W. Cooper (King's), (5) A. W. Maynard (H.C. H. and H.), and (6) G. E. Mould (St. Mary's). The following also ran: P. G. Mould, W. H. Orr, R. J. Green, E. L. Payne, W. Davis, E. Morgan, and J. L. Morton.

### EVENTS FOR THE MONTH OF NOVEMBER.

[Items intended for this column must reach the office, 140, Strand, W.C., not later than first post on Monday mornings.]

**November Meetings and Athletics.**

For list of Students' Medical Societies' Meetings and Football Matches during November, see The Hospital for Nov. 1st, p. 84.

### GENERAL CHARITIES.

[This Column is devoted to an account of work of charitable institutions other than Medical Charities. The Editor will be glad to receive reports or news of work at 140, Strand. *Philanthropy* should be plainly written on the envelope.]

The second of the two annual elections of pensioners of the **ROYAL BLIND PENSION SOCIETY OF THE UNITED KINGDOM** takes place on Tuesday, the 25th inst., when it is expected 24 pensioners will be added to the number on the funds, which already exceeds 550. Subscriptions and donations may be sent to Mr. W. Elliott Terry, secretary, 235, Southwark Bridge Road, S.E.

The Rev. Prebendary Whittington will preside at the annual meeting of **THE NATIONAL BENEVOLENT INSTITUTION** at Freemasons' Tavern, Great Queen Street, which is to be held at twelve o'clock on the 27th inst. At this meeting twenty pensioners will be elected, to whom the two candidates whose names have been longest on the books will be added. It is customary to give gratuities to a certain number of the unsuccessful candidates. Subscriptions in aid of the work of the society will be thankfully received by Mr. Henry C. Latreille, secretary, 65, Southampton Row, W.C.

Since the establishment of **THE NATIONAL REFUGES FOR HOMELESS AND DESTITUTE CHILDREN** some forty-seven years ago, upwards of 11,500 children have been received into their various establishments. Of this number nearly 11,000 have been sent out into the world to earn their own living. The children who are received into the homes are trained for service in this country or the colonies. In addition to the two homes for girls at Sudbury, near Harrow, and Ealing, and the four homes for boys at Twickenham, Bisley, and Shaftesbury Avenue, the society has two training ships, the "Arethusa" and the "Chichester," which is a tender to the former, and both of which are moored off Greenhithe, Kent. One of the Homes at Bisley is a farm school, and the latest addition to the useful operations of the society is the Home in Shaftesbury Avenue, which was opened just two and a-half years ago. Here provision is made for the reception of one hundred boys who are wholly maintained, educated, and trained to earn their own living, principally as shoemakers, tailors, and carpenters. The erection of this building, combined with the purchasing of the leasehold and freehold land and furnishing, cost the sum of £20,000. Unfortunately the contributions towards this sum have not yet sufficed to enable the managers to pay off the whole amount, and they are now compelled to make an appeal for further contributions to the amount of over £3,000, in order to enable them to get free from debt. Donations will be gratefully acknowledged by Mr. W. E. Hubbard, treasurer, 4, St. Helen's Place, E.C., or by Mr. W. Williams, Shaftesbury House, Shaftesbury Avenue, W.C.

**THE MISSION TO DEEP SEA FISHERMEN** is to be congratulated upon its prosperity. Though its total liabilities at the close of October were £467 more than in 1889, the committee has been able during the past year to carry to the reserve fund the sum of £1,000, and to maintain ten mission ships, as against nine during the previous year. The two legacies, amounting in all to £1,100, which they have been fortunate enough to receive, arrived at an opportune time. The winter months, when such fierce gales rage in the North Sea, are those in which the resources of the Mission are put to the greatest strain. In order to enable the society to meet the constant drain upon their resources, liberal and constant support from the public is wanted. The objects of the mission are as practical as they have hitherto been successful, and its work is as interesting as it is dangerous. The various fleets of fishing vessels in the North Sea and elsewhere are visited by means of smacks and small vessels, with a view in every possible way to promoting temperance, and to disseminating healthy literature amongst the men. The mission has by its strenuous efforts during the past eight years almost swept away the trade of the "copers" or drinking ships which used to infest the North Sea, and an attempt is now being made to extend the work amongst fishermen on the west coast and in other waters. The latest addition to the mission's fleet is a new hospital trawler, which, it is hoped, will be ready for sea next spring; the total cost of this ship is estimated at about £2,300. Towards this the society has received nearly £1,300, a sum of £875 having been contributed by one sailor, so that contributions to the amount of £1,000 are still required to enable it to complete the vessel. Among the other practical operations of the society are the rendering of surgical and medical aid to the toilers of the deep, and supplying them with warm wraps, mittens, mufflers, &c., and the sale of tobacco at a low price and without profit to the association, in the hope of keeping the men from resorting to the "copers." The most useful woollen goods that ladies can send to the mission are fishermen's seaboot stockings, steering gloves, woollen helmets, mittens, mufflers, and old linen and lint. Secretary, Mr. Alexander Gordon, 181, Queen Victoria Street, E.C.

